# The Role of Osteoprotegerin as a Cardioprotective Versus Reactive Inflammatory Marker: the Chicken or the Egg Paradox

**DOI:** 10.4274/balkanmedj.2018.0579

**Published:** 2018-05-29

**Authors:** Flora Özkalaycı, Öykü Gülmez, Betül Uğur-Altun, Seithikurippu Ratnas Pandi-Perumal, Armağan Altun

**Affiliations:** 1 Department of Cardiology, Başkent University İstanbul Hospital, İstanbul, Turkey; 2 Department of Endocrinology and Metabolism, Başkent University İstanbul Hospital, İstanbul, Turkey; 3 Somnogen Canada Inc., College Street, Toronto, Canada

**Keywords:** Cardiovascular disease, heart, inflammatory markers, osteoprotegerin

## Abstract

Cardiovascular disease is one of the most frequent causes of mortality and morbidity worldwide. Several variables have been identified as risk factors for cardiovascular disease. Recently, the role of receptor activator of nuclear factor kappa B, receptor activator of nuclear factor kappa B ligand, and the osteoprotegerin system has been recognized as more important in the pathogenesis of cardiovascular disease. Besides their roles in the regulation of bone resorption, these molecules have been reported to be associated with the pathophysiology of cardiovascular disease. There are conflicting data regarding the impact of osteoprotegerin, a glycoprotein with a regulatory role in the cardiovascular system. The aim of this review is to discuss the current knowledge and the role of osteoprotegerin in cardiovascular disease.

Cardiovascular disease (CVD) is one of the most frequent causes of mortality and morbidity among both men and women worldwide (1). Several studies conducted on CVD pathogenesis have identified several variables as risk factors (2,3,4,5). The complex pathophysiology of CVD is still a point of interest. In recent years, the role of receptor activator of nuclear factor kappa B (RANK), RANK ligand (RANK-L), and the osteoprotegerin (OPG) system has been receiving more research attention in terms of the pathogenesis of CVD. OPG was first described by Simonet et al. (6) as a regulator protein for bone metabolism and vascular calcification, which is produced by several tissues, including smooth muscle cells and the endothelium of the human vasculature (7,8,9,10). In addition to their roles in the regulation of bone resorption in calcium and immunologic reactions, RANK, RANK-L, and the OPG system have also been found to be associated with CVD pathophysiology (7,8,9). Several trials have reported higher OPG plasma levels in patients with coronary artery disease (CAD), peripheral artery disease (PAD), chronic heart failure (CHF), and atrial fibrillation, suggesting the association between OPG and CVD (11,12,13,14,15,16).

## OSTEOPROTEGERIN: STRUCTURE, FUNCTION, AND METABOLISM

**Figure 1:**
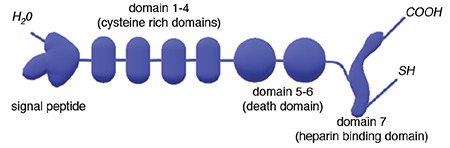
Schematic drawing of the osteoprotogerin molecule.

OPG is a soluble glycoprotein that is synthesized from 401 amino acid residues arranged into 7 structural domains (Figure 1). After losing 21 amino acids, it converts into a mature soluble glycoprotein with 380 amino acids. It is found in the extracellular fluid in the form of either a 60-kDa monomer or a 120-kDa dimer, linked by disulfide bonds. OPG is a member of the tumor necrosis factor (TNF) receptor superfamily and serves as a decoy receptor for RANK-L and TNF-related apoptosis-inducing ligand (TRAIL). OPG is also a key protein for bone metabolism. By binding to RANK-L, OPG inhibits the interaction between RANK and RANK-L, which prevents the osteoclastic differentiation of stromal cells. On the other hand, by binding to TRAIL, it inhibits the apoptosis of transformed cells and tumor cells (6,7,8,9).

Of the 7 structural domains of OPG, domains 1-4 contain cysteine-rich N-terminal amino acids. These domains bind to RANK-L and inhibit osteoclast formation from osteoclast precursors. Domains 5-6 bind to TRAIL and inhibit TRAIL-induced apoptosis. Domain 7 contains the C-terminal amino acid, which is the heparin-binding region of OPG (10,17,18). Although OPG is primarily produced by bone marrow stromal cells, it can also be expressed in dendritic cells and B lymphocytes (19). 1-α 25 hydroxycholecalciferol, interleukin-1 (IL-1), TNF-α, IL-6, IL-7, IL-11, IL-18, calcium and estrogen, transforming growth factor beta, bone morphogenetic protein-2, fibroblast growth factor, angiotensin 2, and platelet-derived growth factor are known to upregulate the expression of OPG, whereas immunosuppressants, parathyroid hormone, glucocorticoids, insulin-like growth factor-1, prostaglandin E2, and peroxisome proliferator-activated receptor gamma ligands downregulate OPG production (20,21,22,23). The tensile force applied on osteoblasts is a mechanical stimulus that also increases OPG synthesis. Moreover, several studies have demonstrated that aerobic exercise is associated with increased OPG levels (24).

Serum OPG levels are associated with its renal clearance. OPG levels increase with the reduction of renal creatinine clearance (25). Further increases can be seen in patients with end-stage renal disease receiving hemodialysis, as OPG monomer cannot be removed through the polysulfone membrane during hemodialysis (25). In addition, OPG level increases with advanced age, especially in the presence of diabetes mellitus (DM) (14,26,27,28,29,30,31). Most of the epidemiologic studies have demonstrated a positive correlation between age and OPG levels (30,31). OPG levels were found to be increased with age in both men and women (30). Premenopausal women had higher OPG levels than men below age 50 years, whereas postmenopausal women had similar OPG serum levels compared with an age-matched male population (30). Moreover, in the Dallas Heart Study, women had higher OPG levels than men, which were compatible with the majority of other epidemiologic studies (32,33). Based on these results, investigators also concluded that gender difference in OPG levels suggests that sex steroids regulate OPG expression *in vivo* (34). Estrogen enhancement of OPG secretion by osteoblastic cells may play a major role in the antiresorptive action of estrogen on bone (35). Khosla et al. (30) demonstrated that OPG levels were positively correlated with bone turnover markers in the male gender. In another study, parenteral administration of OPG prevented bone resorption in postmenopausal women (36). These studies suggest that OPG levels increase concomitantly with bone turnover markers as a compensatory response to enhanced bone resorption process. Moreover, there are several studies indicating that OPG deficiency is primarily associated with osteoporosis and arterial calcification (13,37). In addition, osteoporosis was reported to be accompanied by severe vascular calcification in OPG-deficient mice (7).

### Relationship Between Osteoprotegerin Levels and Cardiovascular Disease

Coronary artery calcification (CAC) is a well-known indicator for atherosclerotic plaque burden. The factors that interfere with CAC also interfere with coronary atherosclerosis and CVD, respectively (38). According to clinical studies, vascular calcium burden is an indicative marker for the severity of atherosclerosis and arteriolosclerosis (39). Several studies have shown that the RANK–RANK-L–OPG system is related to atherosclerotic plaque calcification (40,41).

**Relationship between osteoprotegerin levels and stable coronary artery disease: **Several studies have shown that OPG levels are predictive of CAD (11,12,42,43). Lieb et al. (11) measured OPG levels in 3.250 Framingham Heart Study participants and found a positive association between circulating OPG levels (but not RANK-L) and CVD incidence and mortality. Some investigators have demonstrated a positive correlation between circulating OPG levels and the severity of atherosclerosis and the number of diseased vessels, especially in patients with type 2 DM (12,26,42). Ghaffari et al. (43) reported a significant relationship between serum OPG levels and coronary artery stenosis. Furthermore, they found that major adverse cardiovascular events occurred more often in patients with higher baseline OPG levels. Kiechl et al. (44) concluded that OPG was an independent risk factor for the onset of CVD and progression of atherosclerosis. In addition, they found that OPG was independently and significantly related to incident CVD and vascular mortality (44). Poornima et al. (45) showed that in postmenopausal women, the higher OPG levels were associated with higher CAC. In a study comparing OPG levels between patients with cardiac syndrome X (CSX), healthy obese subjects, and healthy lean subjects, it was found that OPG levels were significantly lower in patients with CSX than those in healthy lean subjects. The authors concluded that low OPG levels might play a role in systemic microvascular abnormalities observed in patients with CSX (46). OPG levels were found to be indicative of cardiovascular morbidity and mortality in patients with chronic kidney disease (47,48,49).

**Relationship between osteoprotegerin levels and Acute Coronary syndrome: **In patients with ST-elevation myocardial infarction (STEMI) who underwent primary percutaneous coronary intervention, the OPG levels were found to be significantly associated with worse long-term cardiac outcomes (50). Jansson et al. (51) showed that OPG was an independent predictor for long-term mortality and CHF in patients with unstable angina pectoris (USAP). Moreover, Luo et al. (52) investigated OPG and OPG/RANK-L levels in patients with stable angina pectoris (SAP) and Acute Coronary syndrome (ACS). They found that serum OPG and OPG/RANK-L levels were significantly higher in the ACS group than in the SAP group. In the control group, which consisted of healhy subjects, the OPG and OPG/RANK-L levels were significantly lower than those of both the ACS and the SAP groups (52). However, in another study involving patients with SAP, USAP, and non-STEMI, the OPG levels showed no correlation with the severity of CAD, which might be due to OPG and/or RANK-L polymorphism and some other possible genetic variations, but it may be accepted as an indicator of coronary atherosclerosis (53). Higher OPG levels detected in patients with ACS might be associated with increased cytokine levels. This increase may be a proatherogenic pathway for further inflammation. Zauli et al. (54) showed that endogenously released OPG in response to TNF-α promoted an increased expression of adhesion molecules and leukocyte–endothelial cell interaction, which aggravates inflammation.

### Relationship Between Osteoprotegerin Levels and Peripheral Artery Disease

Multiple studies that had been carried out in various patient groups to evaluate the association between OPG and PAD found significant correlations (55,56,57). OPG levels appear to predict PAD in several patient groups. Lee et al. (56) demonstrated a positive correlation between OPG levels and cardio-ankle vascular index, which predicts atherosclerosis in hypertensive patients, thus suggesting arterial stiffness. In a study performed in patients with type 2 DM, serum OPG levels were higher in those with PAD than in those without PAD (57). In another study involving patients with nonalcoholic fatty liver disease (NAFLD), the OPG level was found to be higher in patients who had lower aortic flow propagation velocity and higher epicardial fat thickness (58).

### Relationship Between Osteoprotegerin Levels and Stroke

Several studies have demonstrated higher OPG levels in patients with atherosclerotic cerebrovascular disease than those in healthy subjects (44,59,60). Carotid intima-media thickness (CIMT) is one of the risk factors predicting stroke and CVD (61,62). Kiechl et al. (44) also showed that OPG levels were associated with the severity and progression of carotid artery disease. Patients with chronic kidney disease and patients with increased OPG levels had significantly higher CIMT values than those in subjects with lower OPG levels (47). In women with gestational DM, increased serum OPG levels showed a positive correlation with CIMT (59). There are conflicting data regarding the association between stroke severity and OPG levels. In one study, no association was found between the risk of ischemic stroke and OPG levels (63). However, another study reported that plasma OPG levels were higher in patients with severe stroke (64). Mogelvang et al. (60) evaluated the association between plasma OPG and high sensitive C-reactive protein (CRP) levels and hospitalization for ischemic stroke, ischemic heart disease (IHD), and all-cause mortality. They found that OPG levels were an independent predictor of combined end-points of ischemic stroke, IHD, and all-cause mortality. Along with the predictive value of OPG in ischemic stroke, Guldiken et al. (65) demonstrated that OPG was also associated with stroke subtypes.

### Relationship Between Osteoprotegerin Levels and Chronic Heart Failure

OPG is a secretory glycoprotein that may exert a compensatory response to increased inflammatory activity in patients with CHF. Omland et al. (66) assessed the relationship between OPG levels and left ventricular (LV) function in patients who were enrolled in the Dallas Heart study. High levels of OPG were found to be associated with increased LV end-systolic volume and decreased LV ejection fraction (66). After adjusting for potential confounders in sex-specific multivariable models, OPG levels were found to be positively correlated with LV thickness, mass, and LV concentricity index in men, but not in women (66). In another study, serum OPG levels were predictive of CHF development in patients with ACS, independent of conventional risk markers such as troponin I, CRP, B-type natriuretic peptide, and ejection fraction (67). Moreover, CHF was shown to be associated with increased OPG–RANK–RANK-L axis, indicating inflammation characterized by matrix degradation and remodeling of the myocardial tissue (16).

### Relationship Between Osteoprotegerin Levels and Valvular Heart Disease

Valvular heart disease is associated with inflammation and calcification of valve tissue. Proinflammatory cytokines activate the endothelial cells and increase OPG release (68). It was shown that increased OPG levels were associated with decreased osteoclastic differentiation in stenosis of aortic valves (69). Moreover, calcification of aortic valves in the elderly has been attributed to the inflammatory milieu, which promotes the osteogenic transformation of valvular cells (69,70). LDLr ˉ/ˉ mice expressing only apolipoprotein B100 were prone to develop aortic valve calcification. Weiss et al. (71) investigated the impact of exogenous OPG on aortic valve calcification in hypercholesterolemic LDLr ˉ/ˉ Apob100/100 mice and found that exogen OPG attenuates osteogenic transformation of valve cells and bone-like matrix synthesis, indicating that the aortic valve function was protected by the OPG treatment (71).

### Relationship Between Osteoprotegerin Levels and Hypertension

The renin–angiotensin–aldosterone system is one of the hallmarks in blood pressure regulation. Some studies have demonstrated the indirect effects of angiotensin II on the activation of osteoclasts via the activation of RANK-L gene expression in osteoblasts (72,73). Based on these data, it can be speculated that blood pressure and OPG may have an interactive relationship. It has been shown that angiotensin II blockage downregulates OPG levels *in vitro*, suggesting the possible link between high OPG levels and increased blood pressure (74). It may be suggested that increased OPG levels might be a protective response to vascular stiffness observed in hypertensive subjects. Wang et al. (75) showed that patients with higher carotid–femoral pulse wave velocity measurement, which is a gold standard for assessing arterial stiffness (76), had higher OPG levels. In the Dallas Heart study, higher OPG levels were also associated with hypertension (32). Browner et al. (14) showed that OPG levels were slightly higher in women with high blood pressure than in those with normal blood pressure, especially in patients aged ≥65 years (14). These results may be attributed to the relationship between OPG levels, arterial stiffness, and inflammation. Hypertension and arterial stiffness associated with vascular calcification and decreased elasticity may increase inflammation and expression on OPG in order to decrease the vascular stiffness (33). In several studies, the association between OPG levels and blood pressure was assessed along with other cardiovascular risk factors. Akyuz et al. (77) compared the OPG levels of resistant and nonresistant hypertension in patients with Obstructive Sleep Apnea syndrome. They also assessed the relationship between CIMT, apnea–hypopnea index (AHI), and OPG levels among the study groups. They found that OPG levels were higher in patients with resistant hypertension than in patients with nonresistant hypertension (77). Moreover, they found no relationship between AHI and OPG levels but showed a positive correlation between CIMT and OPG levels in resistant hypertensive patients. However, this finding did not suggest a direct cause-and-effect relationship between higher OPG levels and high blood pressure (77). In contrast to these findings, no significant relationships were found between blood pressure and OPG levels in a Korean women study that was performed in premenopausal and postmenopausal women (78). The difference in the results between these two studies might be due to the different characteristics of the study participants and the different assays used to measure OPG levels (77,78).

### Relationship Between Osteoprotegerin Levels and Diabetes Mellitus

Numerous studies have shown that serum OPG levels were higher in patients with DM than in those without DM (28,14,79). Different investigators have reported that serum OPG levels were increased in patients with type 1 and type 2 DM (28,80,81). OPG levels were also found to be higher in patients with longer duration of DM. Furthermore, in patients with DM and CVD, serum OPG levels were found to be higher than those in patients with DM and without CVD (81,82). OPG levels were shown to predict subclinical atherosclerosis and near-term cardiovascular events in uncomplicated type 2 DM (42). Diabetic end-organ damage was more likely to be observed in patients with higher OPG levels (28). In a study designed to determine whether OPG could be a marker for nephropathy in type 1 DM, OPG levels were found to be high only in patients with nephropathy; however, there was no significant relationship between other microvascular and macrovascular complications and OPG levels (83). Experimental studies have shown that increased OPG levels in patients with DM were related to proinflammatory cytokines such as TNF-α and IL-6 rather than high serum glucose levels (84). These data indicate that the increased OPG levels in patients with DM are related to proinflammatory cytokines but are not related to insulin and high glucose levels (79,84,85).

### Relationship Between Osteoprotegerin Levels and Obesity

There is still a conflict of data regarding the relationship between obesity and serum OPG levels, which might be attributed to the difference between the sample groups and methods, such as comorbidities and demographic features of patients of each study and the assays used for measuring OPG levels. Gannage-Yared et al. (86) found no correlation between body mass index and OPG levels, but they showed a positive correlation between insulin sensitivity and CRP levels and OPG levels in obese patients, suggesting that the inflammatory process in obesity promotes OPG increase. Ashley et al. (24) designed a study to examine the relationship between OPG levels, obesity, and insulin sensitivity in a healthy population. OPG levels were found to be significantly lower in obese subjects than in normal-weight and overweight subjects. In addition, a positive relationship was found between OPG levels and insulin sensitivity among all subjects (24). Consistent with these findings, a study involving obese, healthy, and lean subjects found that OPG levels were significantly lower in the obese group than in the lean controls (87). Furthermore, lower OPG levels were found in obese subjects with elevated insulin resistance than those found in lean subjects and in obese subjects with low insulin resistance (87). The relation between circulating OPG and insulin resistance assessed HOMA-IR was in investigated at obese and normal-weight women in premenopausal period (88). OPG showed a negative correlation with insulin and HOMA-IR in premenopausal obese women (88). According to these results, it may be suggested that increased insulin levels are associated with decreased OPG levels. The mechanism of decreased OPG levels in insulin-resistant obese subjects remains unclear. Several studies have shown that lower OPG levels observed in obese subjects are attributed to the possible inhibitory effect of insulin on OPG expression (89,90). In an experimental study designed with 9 healthy male subjects, it was shown that acute hyperglycemia did not increase OPG levels in nondiabetic subjects, whereas hyperinsulinemia inhibited OPG expression (89). This finding might be attributed to a possible inhibitory effect of insulin on OPG expression and explain the decreased OPG levels in healthy obese subjects.

### Relationship Between Osteoprotegerin Levels and Metabolic Syndrome

Considering that the Metabolic syndrome (MetS) is a state of obesity, insulin resistance, and inflammation, increased white adipose tissue (WAT) in obese subjects has become a much important issue (91,92). WAT acts as an endocrine organ that promotes inflammation due to its macrophage-rich content (93,94). As inflammation promotes OPG expression, it can be suggested that OPG levels are accepted to be higher in patients with MetS. On the other hand, there are conflicting data regarding the relationship between OPG levels and MetS. According to animal studies, OPG levels were found to be increased in mice fed with a high-fat diet (95). Bernardi et al. (95) reported higher OPG levels in newly diagnosed MetS group than in healthy subjects after adjusting for other risk factors, which might be due to the inflammatory milieu in MetS causing increased OPG expression. However, some investigators suggest that there is no correlation between MetS and serum OPG levels (86). The different results may be attributed to the different study groups differing in terms of variables such as sex, age, comorbidities, and diagnostic criteria of MetS.

### Relationship Between Osteoprotegerin Levels and Hyperlipidemia

The relationship between lipid profile and OPG levels is still a matter of debate. Lipid profile has been the subject of few studies, and controversial reports have been published (15, 26,96). The different results obtained are attributed to different study populations. In Korean healthy female subjects, higher total cholesterol and low-density lipoprotein (LDL) levels were associated with higher OPG levels (78). In the Dallas Heart study (32), the participants’ higher OPG levels were found to be associated with hypercholesterolemia. In contrast, Gannage-Yared et al. (86) found no correlation between OPG levels and lipid profile in obese subjects. These studies suggest that the increased OPG expression is a protective response to elevated LDL levels that promote the atherosclerotic process. Moreover, it can be speculated that higher OPG levels in hypercholesterolemia are a result of the inflammatory milieu caused by increased LDL and total cholesterol levels. LDL-lowering therapy was shown to have conflicting results in patients with type 2 DM. Although simvastatin treatment reduced OPG levels (96), lovastatin and pravastatin treatment increased the plasma levels of OPG (97,98).

## ROLE OF OSTEOPROTEGERIN AS A CARDIOPROTECTIVE VERSUS REACTIVE INFLAMMATORY MARKER: THE CHICKEN OR THE EGG PARADOX

Recent studies have demonstrated that serum OPG levels are a strong predictor of cardiovascular mortality and morbidity (11,12). In a systematic review performed by Hosbond et al. (99), the authors evaluated the relationship between OPG and CAD, ACS, PAD, and cerebrovascular disease. They analyzed 14 studies with clearly defined cohorts and found that OPG levels are associated with the presence and severity of stable CAD, ACS, and cerebrovascular disease, but not associated with PAD. The atherosclerotic process involves mechanical and immunological mechanisms, and arterial calcification is an important indicator of atherosclerotic plaque burden. According to recent studies, in contrast to our previous knowledge, vascular calcification is an actively regulated process and human vascular smooth muscle cells can express osteoblastic transcription factors (100,101) and bone-regulating proteins such as matrix Gla protein, osteopontin, osteocalcin, collagen 1, osteonectin, bone morphogenic proteins, alkaline phosphatase, and bone sialoprotein. The calcification can occur either in an intimal or in a medial layer of arteries. Remodeling of the arterial wall includes degeneration of elastin fibers, increase in collagen fibers, and thickening of the arterial wall (102,103). As a result, calcification of elastic components of the medial layer increases the arterial wall thickness, thus causing arterial stiffness. Increased arterial stiffness causes arterial shear stress on the vascular wall and hence develops a tendency to atherosclerosis (104). Based on these data, it can be speculated that OPG is a protective mediator for atherosclerosis due to the inhibition of vascular calcification.

Atherosclerosis is also an immunological process. In an experimental study performed on unstable coronary plaques, increased expression levels of RANK-L from T cells and RANK from monocyte series were demonstrated by Shaker et al. (105). Therefore, it has been suggested that the increase in RANK-L is related to the increase in leukocyte response and matrix degradation, thus causing the inflammatory response. According to this conclusion, the interaction between RANK and RANK-L is attributed to plaque destabilization, and it can be speculated that as a decoy receptor for RANK-L, OPG behaves as an anti-inflammatory mediator (105). There are various studies that proclaim the angiogenetic and favorable effects of the interaction between RANK and RANK-L on endothelial cell survival (106,107,108). Seccihiero et al. (109) demonstrated that adding RANK-L into cell cultures, regardless of OPG presence, showed protective effects on endothelial cells by activating intracellular pathways, which results in nitric oxide synthesis. In another study, increased OPG levels were found in the presence of TNF-α, which can induce leukocyte adhesion molecule expression from endothelial cells and cause progression of atherogenesis (54).

OPG is a decoy receptor for TRAIL, which is attributed to TRAIL-associated apoptosis. TRAIL levels were found to have a negative correlation with CRP (110). Some researchers have concluded that by inhibiting TRAIL interaction with its receptor, OPG inhibits apoptosis and thereby intrudes the cellular barrier between the vessel lumen and plaque. In contrast, some researchers claim that OPG could increase leukocyte adhesion to endothelial cells both *in vitro* and *in vivo* with regard to the OPG’s heparin-binding region (54). Based on these data, it can be concluded that OPG behaves as a proinflammatory mediator for endothelial cells (54). Another explanation that indicates the proinflammatory behavior of OPG is by inhibiting TRAIL. Administration of TRAIL was shown to exhibit an anti-atherosclerotic activity in apolipoprotein E-null diabetic mice (109). Therefore, as a decoy receptor for TRAIL, OPG might serve in favor of atherogenesis.

The conflicting results reported by different studies may be attributed to the different ELISA kits used for measuring OPG levels. OPG measurements can be done in both plasma and serum; therefore, caution is needed when comparing OPG levels from different studies due to the differences among sample types (111). OPG gene polymorphisms (112) may be another explanation for the divergent results in different studies.

No consensus is yet available on the function of OPG, but all these studies have concluded that OPG is a promising biomarker for CAD, regardless of whether it plays a protective role against atherosclerosis or causes atherosclerosis with its proatherogenic features. Further study is needed to define its role in pathogenesis and in the process of CVD.

## Figures and Tables

**Table 1:**
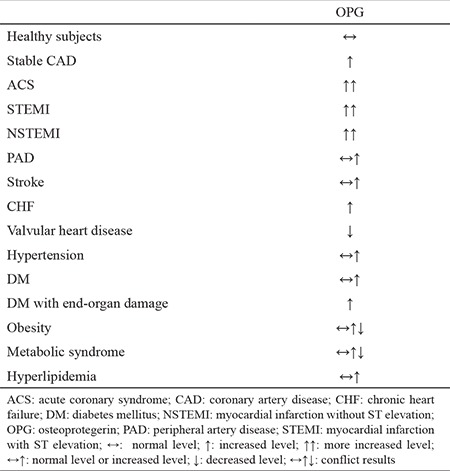
Comparison of serum osteoprotegerin levels in different patient populations
